# Overpayment for Generic Drugs Under Medicare Part D

**DOI:** 10.1001/jamahealthforum.2025.0012

**Published:** 2025-02-28

**Authors:** Inmaculada Hernandez, Nico Gabriel, Yuvraj Pathak, Ryan N. Hansen, Sean D. Sullivan

**Affiliations:** 1 Division of Clinical Pharmacy, Skaggs School of Pharmacy and Pharmaceutical Sciences, University of California, San Diego, La Jolla; 2 West Health Policy Center, Washington, DC; 3 The CHOICE Institute, School of Pharmacy, University of Washington, Seattle

## Abstract

This cross-sectional study evaluates whether certain pharmacy benefit manager practices result in increased out-of-pocket costs for Medicare Part D beneficiaries.

## Introduction

Generic drugs represent 90% of prescriptions filled in the US.^1^ Prior research suggests that, for certain generic drugs, pharmacy benefit managers (PBMs) provide unjustifiably high point-of-sale reimbursement to pharmacies.^2^ For instance, while generic imatinib costs $126 per 30-day supply, PBMs reimbursed at an average of $3780, or 30 times acquisition costs.^2^ We evaluated whether these reimbursement practices result in increased out-of-pocket costs for Medicare Part D beneficiaries.

## Methods

This cross-sectional study was approved by the University of California, San Diego, and Advarra institutional review boards. Informed consent was not sought because data were deidentified. The STROBE reporting guideline was followed.

We analyzed 2021 claims from Medicare Part D beneficiaries for the top 50 generic drugs by spending (eMethods, eTable 1, and eFigure in Supplement 1).^2^ We evaluated claims filled by beneficiaries enrolled in stand-alone prescription drug plans (PDPs) offered by the top 6 Part D sponsors, which account for 90% of stand-alone Part D enrollment (eTable 2 in Supplement 1).^3^ We restricted sampling to claims with no low-income subsidy and that were filled in the initial coverage or coverage gap phase, in which beneficiaries are responsible for 25% of costs. For each claim, we calculated the total reimbursement and patient liability. Patient liability was the sum of patient payment amount and other true out-of-pocket costs (payments made by third-party payers, including state pharmacy assistance programs or charities).

We compared total reimbursement and patient liability to drug acquisition costs, estimated using the National Average Drug Acquisition Cost. We standardized outcomes per 30-day supply equivalents and reported means and medians across Part D sponsors. We also report results for a subset of products with extreme reimbursement rates, defined as products reimbursed by at least 1 Part D sponsor at an average of more than 10 times acquisition cost and with an average reimbursement exceeding acquisition cost by at least $50 per 30-day supply (eTable 3 in Supplement 1).^2^

## Results

### Top 50 Generic Drugs

The PDP plan administered by Elixir Insurance, owned by Rite Aid Corporation, reimbursed generics at a mean (SD) of $22.46 ($123.15) and a median (IQR) of $10.57 ($12.61) above acquisition cost (Table). Mean patient liability exceeded drug acquisition costs for all Part D sponsors except for Humana.

**Table.** Average Reimbursement and Patient Liability Rates in 2021, by Part D Sponsor

Medicare Part D sponsor	30-d Supply equivalents, $									
	Drug acquisition cost^a^		Reimbursement^b^		Reimbursement − acquisition cost		Patient liability^c^		Patient liability − acquisition cost	
	Mean (SD)	Median (IQR)	Mean (SD)	Median (IQR)	Mean (SD)	Median (IQR)	Mean (SD)	Median (IQR)	Mean (SD)	Median (IQR)
**Top 50 generics**										
Centene	5.88 (16.08)	2.34 (3.26)	19.88 (110.16)	9.58 (13.13)	13.99 (104.57)	6.37 (10.34)	9.36 (29.93)	3.35 (7.25)	3.48 (25.64)	0.13 (5.02)
Cigna	6.59 (18.43)	2.49 (3.50)	16.39 (74.29)	8.51 (10.68)	9.80 (67.18)	5.34 (9.10)	8.30 (23.94)	3.00 (7.73)	1.71 (21.08)	0.07 (6.07)
CVS	5.46 (12.10)	2.34 (3.33)	16.93 (107.51)	5.60 (11.50)	11.47 (102.38)	2.91 (9.38)	10.47 (29.77)	4.80 (10.06)	5.01 (24.83)	0.50 (6.23)
Humana	6.95 (20.08)	2.59 (3.63)	13.11 (90.86)	4.66 (6.69)	6.17 (80.23)	1.63 (2.98)	5.40 (24.00)	2.02 (3.43)	−1.55 (20.18)	−0.70 (3.59)
Rite Aid	4.23 (10.16)	2.25 (3.05)	22.46 (123.15)	10.57 (12.61)	18.22 (117.67)	7.61 (10.77)	6.47 (31.11)	1.85 (4.87)	2.24 (26.41)	−0.06 (2.76)
UnitedHealth	6.93 (19.27)	2.54 (3.50)	18.49 (30.75)	11.41 (8.76)	11.56 (17.76)	9.09 (8.47)	9.41 (13.73)	5.12 (10.53)	2.47 (16.01)	2.04 (8.29)
**13 Products with extreme reimbursement rates^d^**										
Centene	9.31 (22.01)	5.06 (1.58)	74.43 (401.51)	45.04 (30.02)	65.11 (385.66)	38.81 (31.08)	32.64 (95.23)	20.17 (35.75)	23.32 (81.23)	13.23 (39.94)
Cigna	8.34 (18.67)	5.09 (1.58)	42.96 (228.99)	20.38 (10.41)	34.62 (214.54)	14.11 (9.41)	20.54 (62.43)	13.50 (19.25)	12.20 (49.22)	7.41 (17.68)
CVS	8.84 (25.94)	4.82 (1.47)	85.10 (399.50)	46.08 (22.61)	76.26 (384.48)	40.17 (22.48)	40.53 (95.06)	34.76 (30.73)	31.69 (82.61)	29.86 (30.51)
Humana	10.82 (29.72)	5.06 (1.58)	48.95 (276.64)	9.00 (10.04)	38.13 (250.05)	3.83 (6.83)	15.40 (69.16)	3.76 (6.43)	4.58 (44.00)	−2.02 (5.32)
Rite Aid	9.70 (27.67)	5.09 (1.13)	172.46 (552.12)	83.85 (183.49)	162.76 (531.10)	79.43 (182.44)	46.00 (133.54)	23.13 (39.44)	36.30 (114.34)	17.61 (37.48)
UnitedHealth	10.35 (28.36)	4.82 (1.58)	34.90 (56.62)	21.40 (30.73)	24.55 (39.92)	15.89 (27.94)	21.01 (23.70)	12.22 (27.12)	10.66 (23.43)	6.89 (23.64)

^a^ Estimated as the mean National Average Drug Acquisition Costs for prescriptions reimbursed by the given Medicare Part D sponsor and standardized to 30-day supply equivalents using the mean number of units in a 30-day supply equivalent for each drug. Of note, this amount was estimated across all Part D sponsors, to ensure that differences in outcomes reported per 30-day supply represented differences in reimbursement rates per unit and not differences in number of units (ie, tablets) dispensed.

^b^ Reimbursement rates represent total payment amounts for prescriptions reimbursed by the given Part D sponsor, expressed in dollars and standardized to 30-day supply equivalents.

^c^ Patient liability was estimated as the sum of patient payment amount and other true out-of-pocket costs (payments made by third-party payers such as state pharmacy assistance programs or charities that count toward patient liability for a prescription). Estimates represent means and medians across all prescriptions reimbursed by the given Medicare Part D parent organization, standardized to 30-day supply equivalents.

^d^ Defined as products reimbursed by at least 1 Medicare Part D sponsor at an average of more than 10 times acquisition cost and with an average reimbursement per 30-day supply equivalent exceeding the acquisition cost by at least $50 (product list in eTable 3 in Supplement 1).

### Products With Extreme Reimbursement Rates

Thirteen products with extreme reimbursement were identified, which accounted for 23.8% of the spending but 60.8% of patient cost-sharing above acquisition costs. Extreme reimbursement was particularly common for the plan operated by Rite Aid Corporation, which reimbursed 9 products with a markup exceeding $100, followed by plans operated by CVS Health (Figure). Abiraterone, cinacalcet, imatinib, and sevelamer presented the most extreme reimbursement and patient liability rates. For instance, CVS Health beneficiaries were liable for a markup of $971.60 above acquisition cost for a 30-day supply of imatinib.

**Figure.**
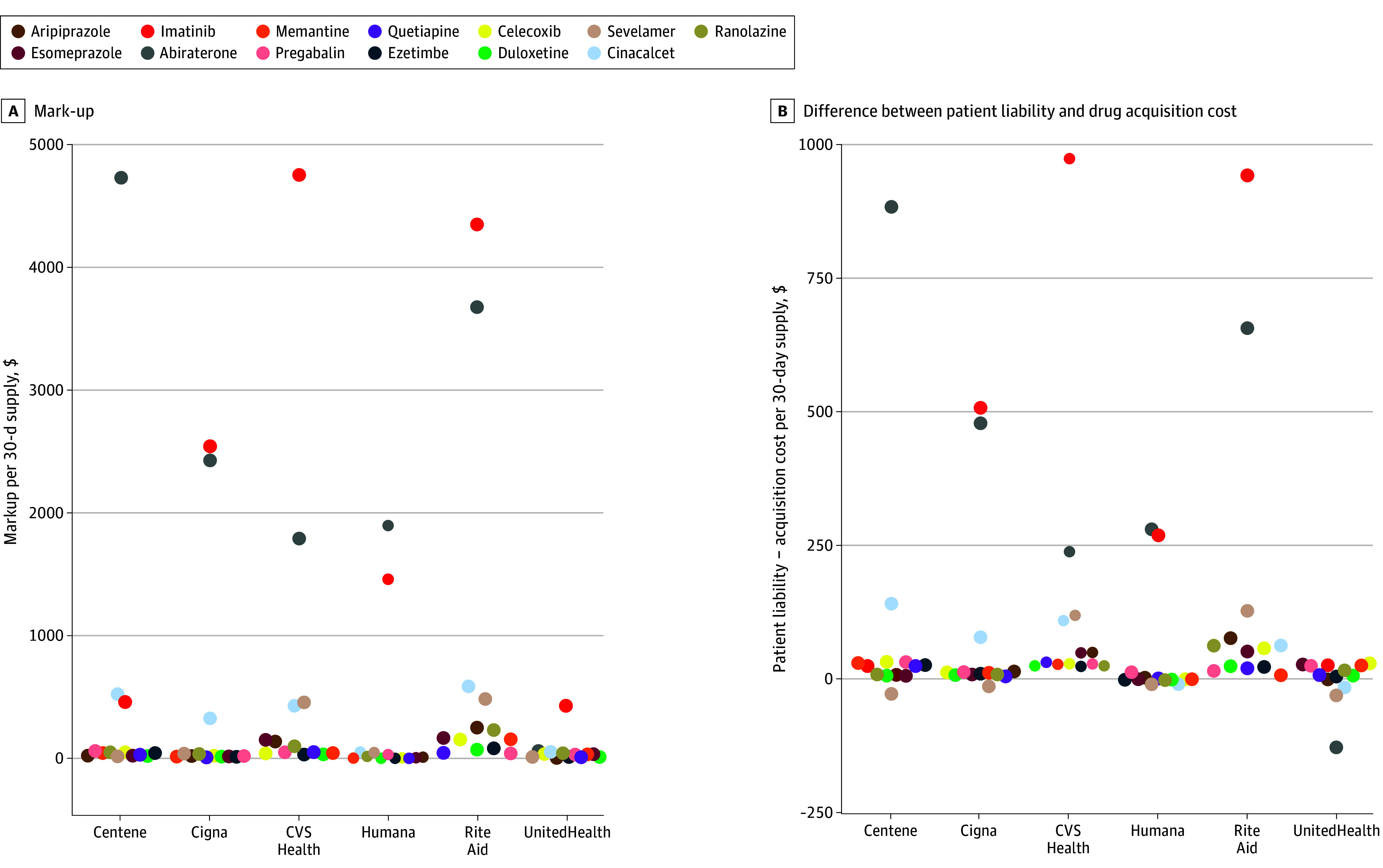
Difference Between Patient Liability and Acquisition Cost per 30-Day Supply A, Mean markup for a generic prescription is estimated as the difference between the reimbursement for a drug and the drug acquisition cost. B, The difference between patient liability and acquisition cost represents the extent to which patients were liable for amounts that exceeded the drug cost. For instance, for imatinib, CVS Health reimbursed pharmacies a mean of $4880 per 30-day supply prescription, which is $4749 above the drug acquisition cost. CVS Health beneficiaries were responsible for $1102 per 30-day prescription, which is $972 above the drug acquisition cost. The figure shows data for the subset of 13 products that had extreme reimbursement rates, defined as products reimbursed by at least 1 Part D sponsor at an average of more than 10 times acquisition cost and with an average reimbursement per 30-day supply equivalent exceeding the acquisition cost by at least $50 (product list in eTable 3 in Supplement 1). Data are shown separately for the top 6 stand-alone Medicare Part D prescription drug plan sponsors.

## Discussion

In this cross-sectional study, the large markups paid by Part D sponsors to pharmacies for selected generic drugs resulted in Medicare beneficiaries being liable for hundreds of dollars above product acquisition cost when using their Part D insurance to fill generic prescriptions. The cost to dispense (estimated at approximately $12/prescription) is unlikely to explain the markups observed, as high as $100 for 9 products and exceeding $1000 for abiraterone and imatinib.^4^

These results are limited by the lack of data to differentiate dispensing fees or posttransaction clawbacks (direct and indirect remuneration fees, eliminated in 2024) and the use of a single year of data. Nevertheless, the results support the passage of legislation to investigate and reform generic reimbursement in Medicare Part D to ensure Medicare beneficiaries access to generic products.^5^ This is critical to avoid more drastic policy solutions with important unintended consequences, such as eliminating generics from insurance coverage, as some groups have proposed to protect patients from overreimbursement practices.^6^

## Notes

Supplement 1. eMethods. Description of the Sample Selection. eTable 1. List of Formulations Included in Analyses eTable 2. List of Part D Contracts Included in Analyses, by Part D Sponsor. eTable 3. List of Products with Extreme Reimbursement Rates and Acquisition Costs. eFigure. Flowchart for Selection of the Sample of Claims. eReferences

Supplement 2. Data Sharing Statement
