# Error in Supplement 1

**DOI:** 10.1001/jamahealthforum.2025.1034

**Published:** 2025-04-11

The Research Letter titled "Overpayment for Generic Drugs Under Medicare Part D,”^1^ published online on February 28, 2025, was corrected to fix the eFigure in Supplement 1. The flowchart for the study’s claim selection process incorrectly stated that claims in the coverage phase were excluded from analysis. Thus, the words “coverage gap” were deleted from the flowchart. This article has been corrected online.
