# Supplementary material for: Overpayment for Generic Drugs Under Medicare Part D
Source: JAMA Health Forum. 2025 Feb 28;6(2):e250012. doi: 10.1001/jamahealthforum.2025.0012 (PMC11871538; doi:10.1001/jamahealthforum.2025.0012)
Supplement: Supplement 1. — eMethods. Description of the Sample Selection. eTable 1. List of Formulations Included in Analyses eTable 2. List of Part D Contracts Included in Analyses, by Part D Sponsor. eTable 3. List of Products with Extreme Reimbursement Rates and Acquisition Costs. eFigure. Flowchart for Selection of the Sample of Claims. eReferences [file jamahealthforum-e250012-s001.pdf]

Supplemental Online Content

Hernandez I, Gabriel N, Pathak Y, Hansen RN, Sullivan SD. Overpayment for generic drugs under Medicare Part D. *JAMA Health Forum.* 2025;6(2):e250012. doi:10.1001/jamahealthforum.2025.0012

eMethods. Description of the Sample Selection

eTable 1. List of Formulations Included in Analyses

eTable 2. List of Part D Contracts Included in Analyses, by Part D Sponsor

eTable 3. List of Products with Extreme Reimbursement Rates and Acquisition Costs

eFigure. Flowchart for Selection of the Sample of Claims

eReferences

This supplemental material has been provided by the authors to give readers additional information about their work.

© 2025 Hernandez I et al. *JAMA Health Forum*.

eMethods. Description of the Sample Selection

We selected the sample of claims in the following steps, as described in eFigure. Using Medicare Part D claims data in 2021 for a 100% sample of Medicare beneficiaries, we first selected claims filled for the top 50 generic drugs in 2021 by Part D spending.[1] We identified the formulation of each generic product with the highest utilization in the sample (listed in eTable 1), and constrained sampling to claims filled for the given formulation. We then identified claims filled by beneficiaries enrolled in Stand-Alone Prescription Drug Plans offered by the 6 Part D sponsors included in analyses (Centene, Cigna, CVS Health, Humana, Rite Aid, and United Health), which combined accounted for 90% of Stand-Alone Part D enrollment.[2] We used pharmacy characteristics data to identify claims dispensed by retail, mail-order, or specialty pharmacies (thus excluding claims filled by institutional pharmacies). Because the objective of the study was to examine cost-sharing, we further constrained sampling to claims filled in either the initial coverage or the coverage gap phase, where beneficiaries are responsible for 25% of generic costs, and for which there were no low-income subsidy contributions. The final sample included 31,880,978 claims filled for the selected formulations of top 50 generic products. We identified 13 products with extreme reimbursement rates. We defined extreme reimbursement as products reimbursed by at least one Part D sponsor at an average of over 10 times acquisition cost and with an average reimbursement per 30-day supply equivalent exceeding the acquisition cost by at least $50. The 10-times threshold was defined following prior studies.[1] The $50 threshold was selected as this is over 4 times the average cost of dispensing, and thus represents a mark-up that is unlikely to be explained by dispensing costs.[5] The subset of claims for drugs with extreme reimbursement rates included 2,630,513 claims for this subset of 13 products (eFigure).

© 2025 Hernandez I et al. *JAMA Health Forum*.

eTable 1. List of Formulations Included in Analyses

Abiraterone Acetate 250mg Tablets
Albuterol Sulfate 90 Mcg HFA Aerosol
Amlodipine Besylate 5mg Tablets
Aripiprazole 5mg Tablets
Atorvastatin Calcium 40mg Tablets
Buprenorphine HCl/Naloxone HCl 8mg-2mg Film
Bupropion HCl 150mg Extended Release Tablets
Carbidopa/Levodopa 25mg-100mg Tablets
Carvedilol 6.25mg Tablets
Celecoxib 200mg Capsules
Cinacalcet HCl 30mg Tablets
Diclofenac Sodium 1 % Gel
Diltiazem HCl 120mg Extended Release Capsules
Duloxetine HCl 60mg Delayed Release Capsule
Esomeprazole Magnesium 40mg Delayed Release Capsules
Estradiol 0.01 % Cream
Ezetimibe 10mg Tablets
Famotidine 20mg Tablets
Fluticasone Propionate 50mcg Suspension Spray
Gabapentin 300mg Capsules
Hydrocodone/Acetaminophen 10mg-325mg Tablets
Hydroxychloroquine Sulfate 200mg Tablets
Imatinib Mesylate 400mg Tablets
Latanoprost 0.005 % Drops
Levetiracetam 500mg Tablets
Levothyroxine Sodium 50mcg Tablets
Lisinopril 20mg Tablets
Losartan Potassium 100mg Tablets
Memantine HCl 10mg Tablets
Mesalamine 1.2g Delayed Release Tablets
Metformin HCl 500mg Tablets
Metoprolol Succinate 25mg Extended Release Tablets
Mirtazapine 15mg Tablets
Montelukast Sodium 10mg Tablets
Nifedipine 30mg Extended Release Tablets
Omeprazole 20mg Delayed Release Capsules
Oxycodone HCl 5mg Tablets
Oxycodone HCl/Acetaminophen 10mg-325mg Tablets
Pantoprazole Sodium 40mg Delayed Release Tablets

© 2025 Hernandez I et al. *JAMA Health Forum*.

eTable 1 cont.

Potassium Chloride 20meq Extended Release Tablets
Pravastatin Sodium 40mg Tablets
Pregabalin 75mg Capsules
Quetiapine Fumarate 25mg Tablets
Ranolazine 500mg Extended Release Tablets
Rosuvastatin Calcium 10mg Tablets
Sevelamer Carbonate 800mg Tablets
Simvastatin 20mg Tablets
Tamsulosin HCl 0.4mg Capsules
Trazodone HCl 50mg Tablets
Venlafaxine HCl 150mg Extended Release Capsules

© 2025 Hernandez I et al. *JAMA Health Forum*.

eTable 2. List of Part D Contracts Included in Analyses, by Part D Sponsor

Part D Sponsor (Parent Organization)	Organization Marketing Name	Contract Number	Enrollment
Centene Corporation	WellCare	S4802	2,580,676
	WellCare	S5768	375,974
	WellCare	S5810	1,214,380
Cigna	Cigna	S5617	993,195
	Express Scripts Medicare	S5660	2,125,032
	Express Scripts Medicare	S5983	81,198
CVS Health Corporation	Aetna Medicare	S5601	5,722,438
Humana Inc.	Humana	S2874	5,560
	Humana	S5552	108,522
	Humana	S5884	3,522,469
Rite Aid Corporation	Elixir Insurance	S7694	827,432
UnitedHealth Group, Inc.	UnitedHealthcare	S5805	142,481
	UnitedHealthcare	S5820	1,883,056
	UnitedHealthcare	S5921	1,885,224
	Optum Insurance of Ohio, Inc.	S8841	687,023
**Total Enrollment**			**22,154,660**

These 15 contracts offered by the 6 selected Part D sponsors account for 90% of enroolment in Stand-Alone Prescription Drug Plans, estimated at 24,419,491 in January 2021.

© 2025 Hernandez I et al. *JAMA Health Forum*.

eTable 3. List of Products with Extreme Reimbursement Rates and Acquisition Costs

Drug Product	Estimated Acquisition Costs per 30-day Supply	Indications
Abiraterone Acetate 250mg Tablets	$252.05	Prostate cancer
Aripiprazole 5mg Tablets	$4.76	Schizophrenia, bipolar disorder, depression, autistic disorder, Tourette's disorder
Celecoxib 200mg Capsules	$6.20	Anti-inflammatory (Osteoarthritis, rheumatoid arthritis, juvenile rheumatoid arthritis, ankylosing spondylitis, acute pain, primary dysmenorrhea)
Cinacalcet HCl 30mg Tablets	$63.15	Hypercalcemia
Duloxetine HCl 60mg Delayed Release Capsule	$4.91	Depression
Esomeprazole Magnesium 40mg Delayed Release Capsule	$8.23	Gastroesophageal reflux disease, risk reduction of NSAID-associated gastric, H. pylori eradication, pathological hypersecretory conditions
Ezetimibe 10mg Tablets	$4.62	Hyperlipidemia
Imatinib Mesylate 400mg Tablets	$127.97	Chronic myeloid leukemia
Memantine HCl 10mg Tablets	$5.12	Dementia
Pregabalin 75mg Capsules	$6.02	Neuropathic pain, postherpetic neuralgia, partial-onset seizures, fibromyalgia
Quetiapine Fumarate 25mg Tablets	$1.55	Schizophrenia, bipolar disorder
Ranolazine 500mg Extended Release Tablet	$20.90	Chronic angina
Sevelamer Carbonate 800mg Tablets	$60.12	Hyperphosphataemia

© 2025 Hernandez I et al. *JAMA Health Forum*.

eFigure. Flowchart for Selection of the Sample of Claims

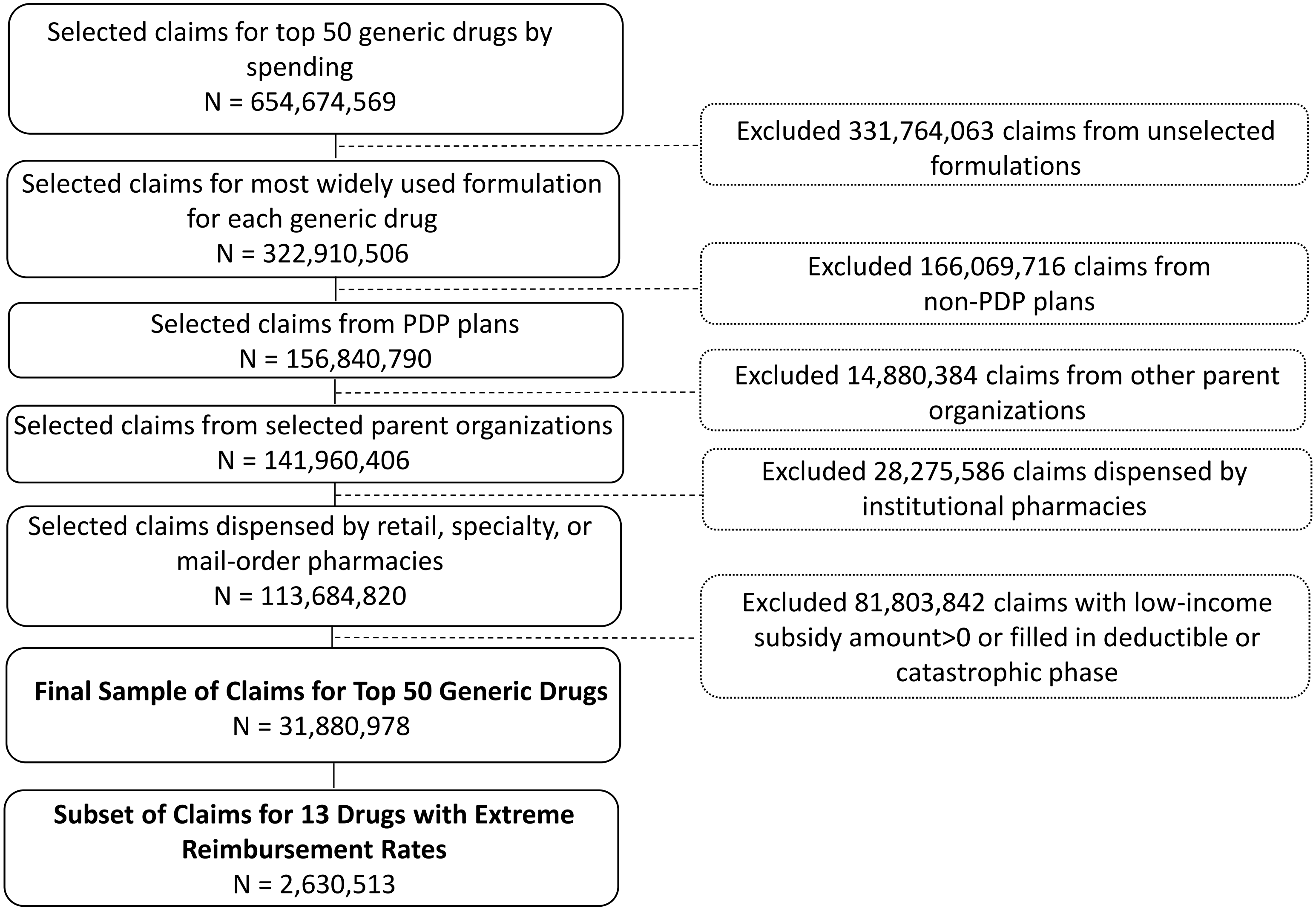

Abbreviations: PDP=Stand-Alone Prescription Drug Plans; NPIs=National Provider Identifiers (referring to dispensing pharmacies in this case).

© 2025 Hernandez I et al. *JAMA Health Forum*.

eReferences

1. Hernandez I, Gabriel N, Kaltenboeck A, Boccuti C, Hansen RN, Sullivan SD. Reimbursement to pharmacies for generic drugs by medicare part D sponsors. *JAMA*. 2023;330(24):2390-2392.

2. Centers for Medicare and Medicaid Services. Medicare Advantage/Part D Contract and Enrollment Data. Accessed March 21, 2023. https://www.cms.gov/research-statistics-data-and-systems/statistics-trends-and-reports/mcradvpartdenroldata

3. National Council for Prescription Drug Programs. DataQ. Accessed August 28, 2022. http://dataq.ncpdp.org/

4. Prescription Drug Plan Formulary, Pharmacy Network, and Pricing Information Files for Order. Accessed March 21, 2023. https://www.cms.gov/Research-Statistics-Data-and-Systems/Files-for-Order/NonIdentifiableDataFiles/PrescriptionDrugPlanFormularyPharmacyNetworkandPricingInformationFiles

5. Shoemaker-Hunt S, McClellan S, Bacon O, Gillis, J, Brinkley J, Schalk M, Olsho L, Taninecz G, Brandt J. Cost of Dispensing Study, January 2020. Abt Associates; 2020. Published online January 2020. https://www.nacds.org/pdfs/pharmacy/2020/NACDS-NASP-NCPA-COD-Report-01-31-2020-Final.pdf

© 2025 Hernandez I et al. *JAMA Health Forum*.
