# Supplementary material for: Overpayment for Generic Drugs Under Medicare Part D
Source: JAMA Health Forum. 2025 Feb 28;6(2):e250012. doi: 10.1001/jamahealthforum.2025.0012 (PMC11871538; doi:10.1001/jamahealthforum.2025.0012)
Supplement: Supplement 2. — Data Sharing Statement [file jamahealthforum-e250012-s002.pdf]

Data Sharing Statement

Hernandez. Overpayment for Generic Drugs Under Medicare Part D. *JAMA Health Forum*. Published February 28, 2025. doi:10.1001/jamahealthforum.2025.0012

Data
Data available: No

Additional Information
Explanation for why data not available: Data were obtained under a data user agreement with CMS that does not allow data sharing
